# Does reduced E-cadherin expression correlate with poor prognosis in patients with upper tract urothelial cell carcinoma? A systematic review and meta-analysis

**DOI:** 10.1097/MD.0000000000017377

**Published:** 2019-10-04

**Authors:** Bum Sik Tae, Chang Wook Jeong, Cheol Kwak, Hyeon Hoe Kim, Ja Hyeon Ku

**Affiliations:** a Department of Urology, Korea University Ansan Hospital,; b Department of Urology, Seoul National University College of Medicine, Seoul National University Hospital, Seoul, Korea.

**Keywords:** e-cadherin, prognosis, survival, urinary tract, urothelial cell carcinoma

## Abstract

**Background:**

E-cadherin has emerged as a prognostic factor of urothelial cell carcinoma. In the present work we investigate the relationship between expression of E-cadherin and clinical outcomes, following radical nephroureterectomy for upper tract urothelial cell carcinoma.

**Methods:**

We systematically searched PubMed, Embase, Cochrane Library, and Web of Science databases to identify eligible studies published until July 2017.

**Result:**

Six studies were included in the meta-analysis, with a total of 1014 patients. The pooled hazard ratio (HR) for recurrence-free survivor was 0.69 (95% confidence interval [CI], 0.44–1.09, *I*^2^ = 63%, *P* = .04). Also, reduced E-cadherin was not significantly associated with poor cancer-specific survivor (pooled HR, 1.40; 95% CI, 0.66–1.43, *I*^2^ = 54%, *P* = .11). The pooled HR for overall survivor was not statistically significant (pooled HR, 0.68; 95% CI, 0.32–1.46, *I*^2^ = 80%, *P* = .007). The results of the Begg and Egger tests suggested that publication bias was not evident in this meta-analysis.

**Conclusions:**

Reduced E-cadherin expression did not appear to be significantly associated with disease prognosis after nephroureterectomy in the meta-analysis. However, further high quality, prospective studies are warranted to better address this issue.

## 1 Introduction

Upper urinary tract urothelial carcinoma (UTUC) is a relatively rare urologic malignancy; however, UTUC has a relatively high recurrence rate and affected patients exhibit poor prognosis, especially those with advanced disease.^[1–3]^ Many researchers have described the survival prognostic factors of UTUC as patient age, pathological factors, and comorbidities.^[1]^ However, previously known predictive factors based on standard clinicopathology findings are insufficient for clinical decisions.^[4,5]^ To overcome this limitation, various biomarkers for urothelial cancers, including centrosome amplification and associated proteins, have been reported.^[6–10]^

E-cadherin is a transmembrane glycoprotein expressed in all normal epithelia and is the prime mediator of cell–cell adhesion.^[11]^ Reduced expression of E-cadherin has been reported as a feature of epithelial–mesenchymal transition (EMT) in epithelial malignancies.^[12,13]^ In previous studies, it was found that transfection of transitional cells with E-cadherin complementary DNA suppresses the invasive potential of the cells; however, abnormal expression of E-cadherin in these cell lines correlates with an aggressive phenotype.^[14,15]^ In addition, several researchers have demonstrated that reduced E-cadherin expression may confer poor prognosis in patients with urothelial bladder cancer (UBC).^[16–18]^ Based on these results, Xie et al reported in their meta-analysis that reduced E-cadherin expression in UBC is associated with poor prognosis and advanced clinicopathological findings.^[19]^

Although decreased expression of E-cadherin is correlated with poor prognosis in patients with UTUC, many studies on E-cadherin have not yet been conducted.^[20–23]^ However, the prognostic value of E-cadherin has not been clearly established in patients with UTUC because UTUC is a relatively rare disease and contradictory conclusions have been reported in previous papers. Thus, we aimed to evaluate the value of E-cadherin as a prognostic factor for UTUC after radical surgery through a systematic review of the literature and meta-analysis.

## 2 Materials and methods

### 2.1 Data sources and search strategy

This study was conducted following the Preferred Reporting Items for Systematic Reviews and Meta-Analyses (PRISMA) statement.^[24]^ The Embase, SCOPUS, and PubMed databases were used to identify potentially relevant published papers. The search was performed on July 30, 2017. The search terms used included “urinary tract,” “cancer,” and “cadherin.” We also carefully checked the references of papers to identify potential additional studies.

### 2.2 Study eligibility

We included papers that met the following criteria in the meta-analysis:

(1) human research;
(2) patients who underwent radical nephroureterectomy (RNU) for UTUC;
(3) studies that assessed E-cadherin protein expression;
(4) availability of Kaplan–Meier/uni- or multivariate Cox proportional hazard model results to estimate the hazard ratios (HRs) and their 95% confidence intervals (CIs).

In addition, we excluded papers with the following criteria:

(1) commentaries, case reports, reviews, letters, and articles that did not provide raw data;
(2) non-English articles;
(3) studies that did not examine E-cadherin expression, clinical parameters, and survival outcomes;
(4) papers that did not present sufficient data for obtaining HRs and 95% CIs;
(5) patients with UTUC who underwent segmentectomy or nephron-sparing surgery.

If there were duplications of study populations or analyses of repeated data, only the largest or most recent article was chosen for meta-analysis.

Initial screening of the relevant articles was performed by 2 reviewers (BST and JHK) based on the titles and abstracts of all the available literature. Two other reviewers (CK and CWJ) independently checked full-text articles to determine whether they met the inclusion criteria of our meta-analysis. If there were discrepancies between the 2 reviewers, discussion with another reviewer (HHK) was undertaken until discrepancies were resolved.

### 2.3 Data extraction and quality assessments

Two reviewers (BST and JHK) independently extracted and crosschecked the required information from all the eligible studies. We extracted the following relevant data: publication data, including author names and the year of publication of the study, study design, origin of the studied population, pathologic UTUC stage, sample size, sex, age of studied patients, cut-off value of E-cadherin expression, follow-up period, and estimated HRs of E-cadherin expression for overall survival (OS), recurrence-free survival (RFS), and cancer-specific survival (CSS), as well as their 95% CIs.

We used the REporting recommendations for tumor MARKer prognostic studies (REMARK) guidelines and quality scales to perform quality assessment. Quality scales and the included study parameters are as follows^[25]^:

(1) sufficient description of the characteristics of the study patients and tumor characteristics, including inclusion and exclusion criteria;
(2) statement of the method of data acquisition, whether prospective or retrospective;
(3) sufficient description of E-cadherin expression measurement;
(4) clear study endpoint;
(5) sufficient description of enrolled patients’ follow-up period;
(6) identification of patients lost to follow-up or not available for statistical analysis. Scores ranged from 0 (studies of the lowest quality) to 8 (studies of the highest quality).

### 2.4 Statistical analysis

We performed a meta-analysis to quantitatively summarize the overall prognostic value of E-cadherin expression. Pooled HRs and 95% CIs were used to examine the effect of reduced E-cadherin expression on the prognosis and clinicopathological features of UTUC, respectively. If the 95% CIs did not overlap, an HR of greater than 1 indicated that reduced expression of E-cadherin was associated with worse prognosis. If explicit survival data were not provided, we calculated survival outcomes based on the available numerical data using methods described by Parmer et al.^[26]^ In addition, if there was an absence or presence of between-study heterogeneity, we used either the fixed effects or the random-effects model. Cochran Q-static and *I*^2^ tests, which describe the percentage of total variation across studies caused by heterogeneity rather than by chance, were used to evaluate heterogeneity between studies. A Q-test with a *P* value of <.05 or an *I*^2^ value of >50% indicated the presence of significant heterogeneity across the selected studies.^[27]^ To estimate potential publication bias, funnel plots, Begg rank correlation test, and Egger linear regression test were used, and a *P* value of <.05 was considered statistically significant.^[28,29]^ All statistical tests were 2-sided, and statistical significance was defined as *P* < .05. We used RevMan 5.0 statistical software (the Cochrane Collaboration, Copenhagen) to perform the meta-analysis. Publication bias was analyzed using R statistical software version 2.13.0 (R Development Core Team, Vienna, Austria; http://www.r-project.org).

## 3 Result

### 3.1 Literature search results

**Figure 1:**
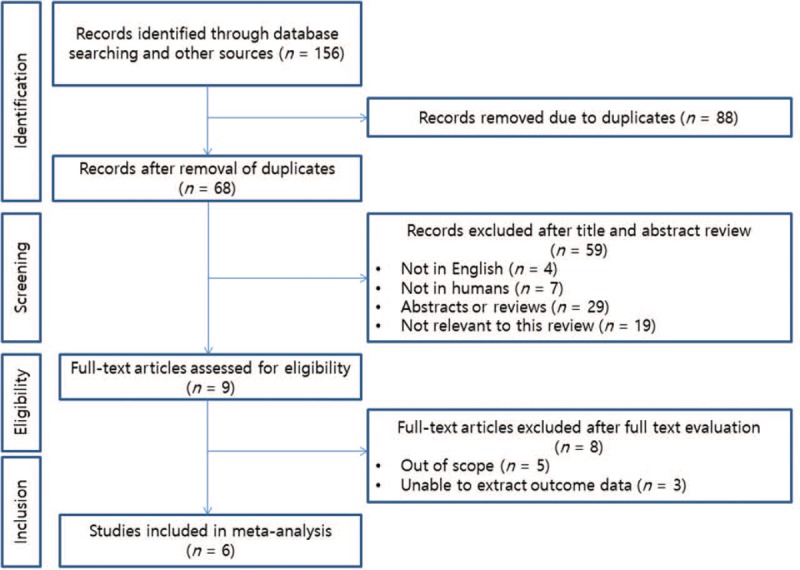
PRISMA statement flow diagram illustrating the search strategy used for the meta-analysis.

Table 1 shows individual data on the characteristics of the selected studies. Three studies were conducted in Japan, 1 study was performed in France, and the remaining studies were multinational from Europe, the USA, and Austria. The studies were published between 2005 and 2017 and the patient recruitment periods ranged from 1981 to 2010. Figure 1 shows the PRISMA flow chart describing the literature search and selection of papers.

**Table 1:**
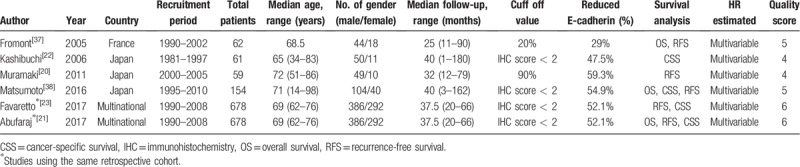
Characteristics of the eligible studies.

### 3.2 Study characteristics

The characteristics of the selected studies are described in Tables 1 and 2. The total number of patients from all the studies was 1014 (individual studies range, 59–678; median, 61). Among the selected studies, Favaretto et al and Abufaraj et al employed the same retrospective cohort of the international UTUC collaboration.^[30]^ All the selected papers reported on retrospective observational cohort studies. For the prognostic value of reduced E-cadherin expression in UTUC, 4, 3, and 3 studies investigated RFS, CSS, and OS, respectively (Tables 3–5, respectively). All the studies evaluated E-cadherin expression using immunohistochemistry (IHC) staining. Reduced E-cadherin expression was defined using different cut-off values in each study; thus, we classified all the patients on the basis of their original studies’ results.

**Table 2:**
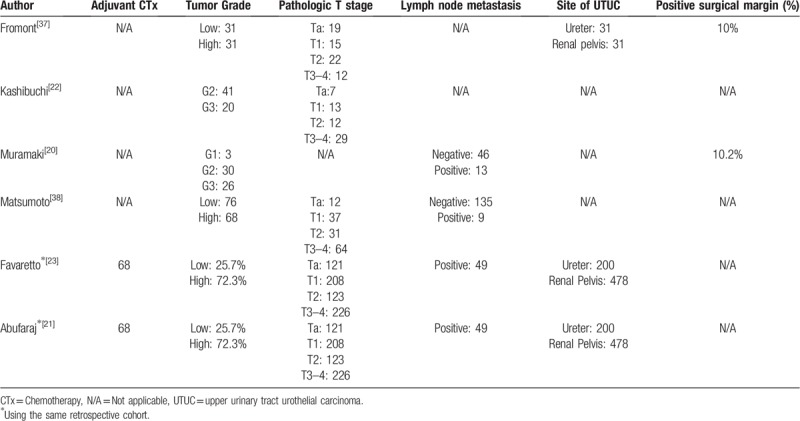
Pathologic characteristics from the eligible studies.

**Table 3:**
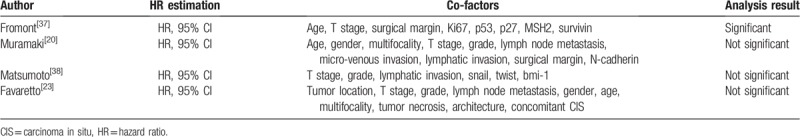
Estimation of the hazard ratio for recurrence-free survival.

**Table 4:**
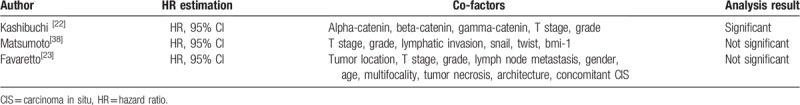
Estimation of the hazard ratio for cancer-specific survival.

**Table 5:**
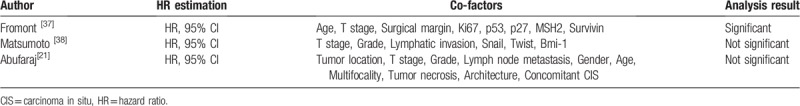
Estimation of the hazard ratio for overall survival.

### 3.3 Outcomes from eligible studies

**Figure 2:**
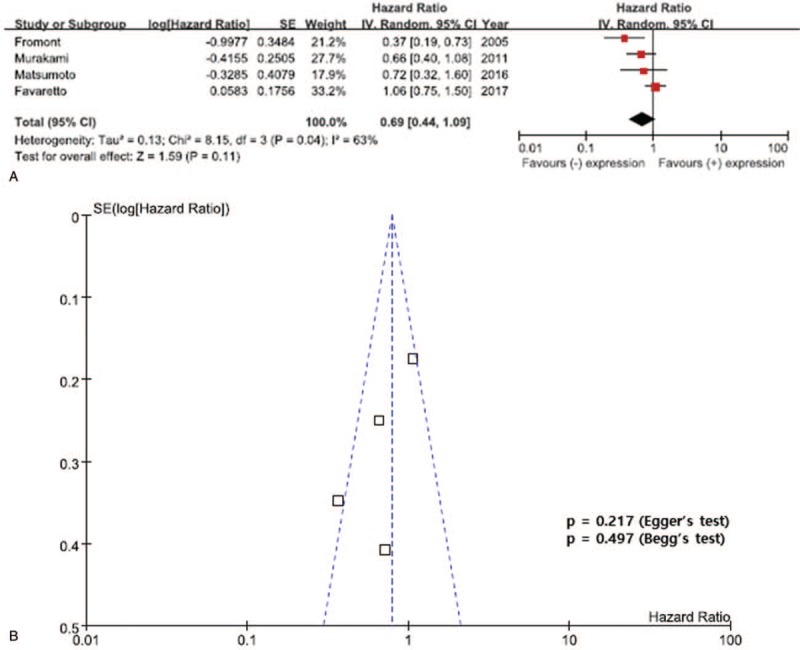
Forest plots of recurrence-free survival (RFS) by reduced E-cadherin. A: The horizontal lines correspond to the study-specific hazard ratios (HRs) and 95% confidence interval (CIs), respectively. The area of the squares reflects the study-specific weights. The diamond represents the pooled results of HRs and 95% CIs. B: The Begg test funnel plots for publication bias. Each point represents a separate study of the indicated association. The vertical line represents the mean effects size.

**Figure 3:**
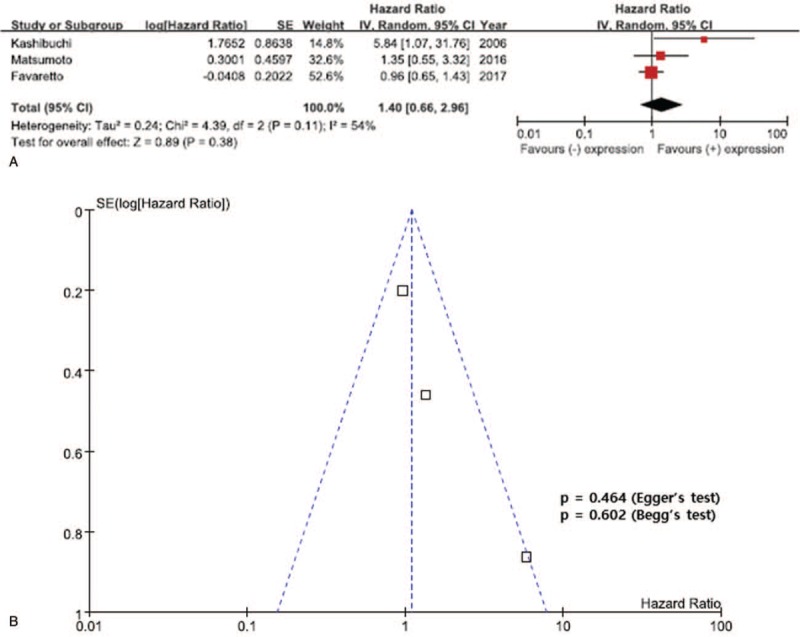
Forest plots of cancer-specific survival (CSS) by reduced E-cadherin. A: The horizontal lines correspond to the study-specific hazard ratios (HRs) and 95% confidence intervals (CIs), respectively. The area of the squares reflects the study-specific weights. The diamond represents the pooled results of HRs and 95% CIs. B: The Begg test funnel plots for publication bias. Each point represents a separate study of the indicated association. The vertical line represents the mean effects size.

**Figure 4:**
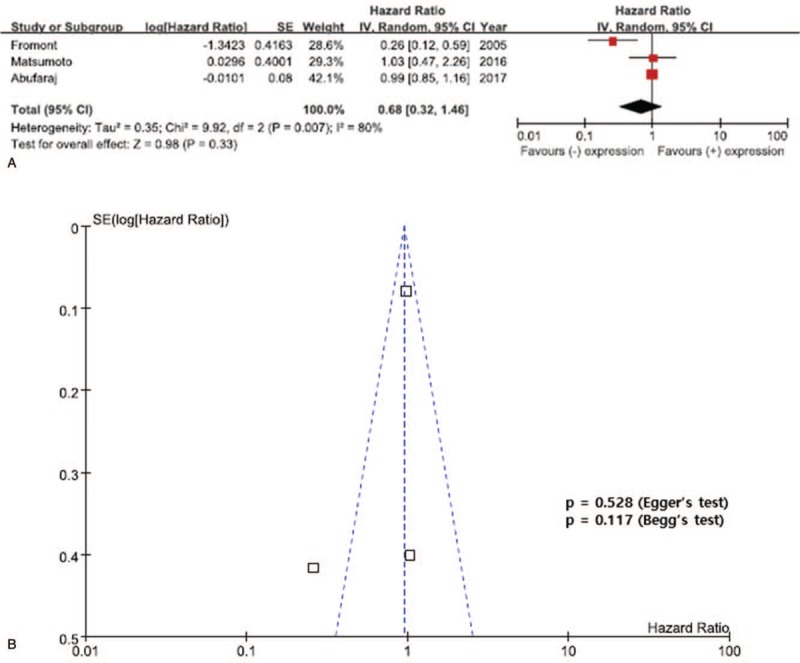
Forest plots of overall survival (OS) by reduced E-cadherin. A: The horizontal lines correspond to the study-specific hazard ratios (HRs) and 95% confidence intervals (CIs), respectively. The area of the squares reflects the study-specific weights. The diamond represents the pooled results of HRs and 95% CIs. B: The Begg test funnel plots for publication bias. Each point represents a separate study of the indicated association. The vertical line represents the mean effects size.

The results of the meta-analysis are presented in Figures 2–4. Overall, the pooled HR for RFS was 0.69 (95% CI, 0.44–1.09), suggesting that reduced E-cadherin expression does not correlate with poor prognosis for UTUC and there was significant heterogeneity between the studies (*I*^2^ = 63%, *P* = .04). In addition, there was no statistically significant association between reduced E-cadherin expression and CSS in patients with UTUC (pooled HR 1.40; 95% CI, 0.66–2.96, *I*^2^ = 54%, *P* = .11) (Fig. 3). In addition, a meta-analysis of the 3 studies evaluating the association of reduced E-cadherin expression and OS found that reduced E-cadherin expression was not significantly associated with worse outcomes, with a pooled HR of 0.68 (95% CI, 0.32–1.16) with significant heterogeneity (*I*^2^ = 80%, *P* = .007) (Fig. 4).

### 3.4 Publication bias

Obvious asymmetry was not evident in the funnel plots of any contrast (Figs. 2–4). Begg test (*P* value) and Egger tests (*P* value, intercept with corresponding 95% CI), as well as funnel plots, were used to assess publication bias in this meta-analysis. All the *P* values for Begg test and Egger test for RFS, CSS, and OS were >.05, presenting statistical evidence of the funnel plots’ symmetry. These results suggested that publication bias was not detected in this meta-analysis.

## 4 Discussion

E-cadherin is a known tumor suppressor that plays a central role in suppressing the invasive phenotype of cancer cells, and many researchers have demonstrated that reduced E-cadherin expression is emerging as a factor of poor prognosis in various types of carcinomas.^[31–33]^ Nevertheless, the biological and clinical roles of the E-cadherin-related pathways in urothelial carcinomas are not yet clearly established. Recently, numerous researchers presented that reduced E-cadherin expression in cancer cells is associated with advanced clinicopathological features and poor outcomes in UBC and UTUC.^[19,20,34]^ These associations can be explained based on the biological role played by E-cadherin as a calcium-dependent glycoprotein that is essential for epithelial tissue integrity.^[23]^ Loss of cell–cell adhesion can result in the detachment of cancer cells with eventual loss of the preventive ability against the invasiveness of human carcinoma cells.^[35]^ In addition, reduced E-cadherin expression is considered as an important hallmark of EMT, through which epithelial cells undergo series of changes in morphology, adhesion, and migratory capacity and transform into cells with mesenchymal characteristics.^[36]^

Consequently, E-cadherin has emerged as a valuable prognostic indicator and potential therapeutic target for urothelial carcinoma. Indeed, a recent meta-analysis presented that reduced E-cadherin expression is associated with poor prognosis and advanced clinicopathological characteristics in UBC.^[19]^ However, the prognostic value of reduced E-cadherin expression in UTUC has not yet been established. Therefore, we performed the current meta-analysis to provide valuable evidence on the association between E-cadherin expression and UTUC prognosis.

To avoid bias caused by the different methods used to evaluate E-cadherin expression, we only included papers that reported on IHC-based evaluation methods in our meta-analysis. Our final analysis included clinical outcomes from 6 eligible studies including a total of 1014 patients with UTUC. Among the eligible studies, studies by Favaretto et al and Abufaraj et al employed the same multicenter retrospective cohort; however, there were differences in the primary endpoints between the 2 studies. Thus, we used the results acquired by Favaretto et al to analyze CSS and RFS and the results acquired by Abufaraj et al to analyze OS. Our findings showed that there was no association between reduced expression of E-cadherin and UTUC prognosis. These findings do not correspond with the results of previous meta-analyses on UBC, which demonstrate that reduction of E-cadherin expression is a prognostic factor.^[19]^

Many researchers have shown their interest in studying the effect of E-cadherin expression on the prognosis of patients with UTUC. Nakanishi et al first presented that reduced E-cadherin expression is associated with higher tumor stage and grade in UTUC.^[34]^ In addition, some study results suggested that reduced E-cadherin expression may be a prognosis factor in UTUC. Fromont et al reported that reduced E-cadherin expression was associated with poor OS and RFS.^[37]^ Kashibuchi et al also demonstrated that reduced E-cadherin expression was an independent predictor of CSS in their multivariate analysis.^[22]^ However, after adjusting for the effects of established prognostic factors in multivariable analyses, more clinical results indicated that E-cadherin expression failed to present any independent prognostic value in patients with UTUC.^[20,21,23,38]^ In addition, even in the study by Fromont et al, reduced E-cadherin expression was not related to higher tumor stage and grade in their multivariate analysis.^[37]^

Although many studies have reported that reduced expression of E-cadherin is associated with adverse clinicopathological features, the reason for the lack of independent prognostic value is presumed to be as follows. First, there was no standardization of the E-cadherin IHC method in each study. The use of different primary antibody sources and different antibody dilution ratios in each study could have resulted in different conclusions. If tissue microarrays with standardized staining protocols and automated scoring systems based on bright-field microscopy imaging coupled with advanced color detection software are developed, they might be of aid to overcome the above limitations.^[23]^ Second, the criteria to define reduced expression of E-cadherin were not standardized among the different studies, which could be a potential cause of heterogeneity. Among the eligible studies, Fromont et al suggested a strict cut-off value, and they suggested that a reduction in E-cadherin expression is associated with OS and RFS in their multivariate study. On the other hand, Muramaki et al used scores according to a classification system derived from the work of Shiozaki et al, in which immunostaining was distinguished as normal or abnormal. Shiozaki et al defined the abnormal staining of E-cadherin as including both negative staining (below 10% of the cells with membranous staining) and heterogeneous staining (between 10% and 90% of the cells with membranous staining).^[39]^ It is hypothesized that the above differences in individual studies might have led to variances in HR estimation leading to significant heterogeneity among studies. In addition, these results suggest that the value of E-cadherin expression as a prognostic factor has been greatly diminished because of the relatively broad standard.

As mentioned above, E-cadherin was first revealed to be an independent marker in a subsequent multivariate analysis in 2005.^[37]^ Conversely, most of the studies published thereafter with larger cohorts failed to demonstrate an independent association between E-cadherin expression and UTUC prognosis after surgery.^[10,34,38,40]^ Although the patients with UTUC had pathophysiology similar to that in patients with UBC, the reason for the current meta-analysis results is presumed as follows. First, UTUC has a relatively low incidence rate, but it is aggressive and affected patients have a relatively poor prognosis.^[41]^ It is possible that E-cadherin may not stand out because patients with UTUC have a poor prognosis due to the strong aggressiveness of the carcinoma. Second, distinct genetic profiles or molecular biology between upper and lower urinary tract tumors may affect the results. For example, Roupret et al reported that microsatellite instability was rarely encountered in UBC, whereas it occurred in more than 15% of sporadic UTUC cases.^[42]^ In addition, single-nucleotide polymorphism variabilities in the rs9642880[T] allele on 8q24 and rs798766[T] allele on 4p16 were associated with disease aggressiveness of UTUC; however, these associations were not found in UBC.^[43]^

Although there is no correlation between E-cadherin expression and UTUC prognosis in this analysis, the contributions of our study are as follows. To the best of our knowledge, our study is the first systematic review and meta-analysis to evaluate the prognostic value of E-cadherin expression with a focus on survival benefits in patients with UTUC, which may help clinicians plan subsequent treatments after radical surgery. Second, in the present meta-analysis, studies reporting HRs of cumulative survival rates were qualitatively summarized using standard meta-analysis techniques. In addition, our study is mainly based on adjusted estimates, but no statistical significance was found for all three endpoints: RFS, CSS, and OS.

Despite the highlights mentioned above, there are still some limitations in our meta-analysis that should be pointed out. First, most of the included studies were of a retrospective design. Second, as mentioned above, the criteria to define normal and reduced expression of E-cadherin were not standardized across different studies, which might be the potential cause of heterogeneity. Thus, a more unified scoring criterion based on IHC methods should be defined in the future.^[19]^ Finally, the pathologic stage of UTUC was not homogenous across different studies included in this analysis, which might be a potential cause of heterogeneity. In addition, with more large-scale prospective studies published in the future, an update is necessary to render more persuasive results.

## 5 Conclusions

Our meta-analysis reveals that reduced E-cadherin expression in UTUC may not correlate with disease prognosis. However, to avoid bias in future studies, we recommend standardization of E-cadherin expression cut-off values and providing HR values with their 95% CIs. Our results should be validated by more comprehensive investigations with prospective, large-population studies.

## Author contributions

**Conceptualization:** Ja Hyeon Ku.

**Data curation:** Bum Sik Tae, Chang Wook Jeong, Hyeon Hoe Kim, Ja Hyeon Ku.

**Formal analysis:** Cheol Kwak, Hyeon Hoe Kim, Ja Hyeon Ku.

**Funding acquisition:** Ja Hyeon Ku.

**Investigation:** Bum Sik Tae, Cheol Kwak, Ja Hyeon Ku.

**Methodology:** Hyeon Hoe Kim, Ja Hyeon Ku.

**Project administration:** Cheol Kwak.

**Supervision:** Bum Sik Tae, Chang Wook Jeong, Cheol Kwak, Hyeon Hoe Kim, Ja Hyeon Ku.

**Validation:** Ja Hyeon Ku.

**Writing – original draft:** Bum Sik Tae.

**Writing – review & editing:** Cheol Kwak, Hyeon Hoe Kim, Ja Hyeon Ku.

Bum Sik Tae orcid: 0000-0003-2963-7366.
